# Respiratory Symptoms and Paper Dust Exposure among Workers in the Paper Industry in Ethiopia: A Comparative Cross-Sectional Study

**DOI:** 10.3390/ijerph21101331

**Published:** 2024-10-08

**Authors:** Ararso Tafese, Abera Kumie, Bente E. Moen, Teferi Abegaz, Wakgari Deressa, Samson Wakuma Abaya, Magne Bråtveit

**Affiliations:** 1 Department of Preventive Medicine, School of Public Health, Addis Ababa University, Addis Ababa P.O. Box 90861000, Ethiopia; ararso.tafese@aau.edu.et (A.T.); abera.kumie@aau.edu.et (A.K.); teferi.abegaz@aau.edu.et (T.A.); wakgari.deressa@aau.edu.et (W.D.); samson.wakuma@aau.edu.et (S.W.A.); 2 Centre for International Health, Department of Global Public Health and Primary Care, University of Bergen, 5009 Bergen, Norway; bente.moen@uib.no; 3 Department of Global Public Health and Primary Care, University of Bergen, 5009 Bergen, Norway

**Keywords:** chronic respiratory symptoms, cumulative dust exposure, paper dust, paper industry workers

## Abstract

Chronic respiratory symptoms are a health concern in the paper industry. This study evaluates the association between personal inhalable paper dust exposure and chronic respiratory symptoms among workers in this industry. In total, 270 workers from the paper industry and 267 from a water bottling factory participated. Chronic respiratory symptoms were assessed using a standardized questionnaire, modified from the American Thoracic Society. A job exposure matrix, based on cross-sectional personal measurements of inhalable paper dust, was used to estimate the exposure–response relationship between cumulative dust exposure and chronic respiratory symptoms. There was a higher prevalence of chronic coughs (27.4% vs. 7.5%), breathlessness (25.6% vs. 11%), coughs with sputum (21.1% vs. 1.1%), and wheezing (25.6% vs. 5%) among paper workers compared to those in the water bottling industry. A Poisson regression analysis revealed that the prevalence ratios for chronic coughs (APR = 3.3 and 95% CI: 2.0–5.4), breathlessness (APR = 2.2 and 95% CI: 1.4–3.4), and wheezing (APR = 4.3 and 95% CI: 2.3–7.7) were significantly higher in paper workers than in water bottling workers. Among paper workers, a significant exposure–response relationship was observed between cumulative dust exposure and chronic coughs after adjusting for age, sex, history of respiratory illnesses, work in other dusty industries, and use of biofuels. As there were only four ever-smokers, smoking was not included in the regression analysis. The results show a significant association between dust exposure and coughing, highlighting the need for control measures to prevent the development of respiratory symptoms among workers.

## 1. Introduction

Respiratory diseases are a major cause of mortality and disability worldwide [1], impacting human life, health, and productivity [2,3]. Work-related lung diseases are among the most common occupational diseases with severe consequences such as death, disability, and absenteeism from work [3,4]. A WHO/ILO joint report estimated that chronic obstructive pulmonary disease (COPD) was the leading cause of work-related deaths globally, accounting for 24% of the burden of disease and injury in the period of 2000–2016 [5].

Paper and paper products are commonly used in daily human activities, particularly for packaging purposes [6]. Their production process, which involves the use of paper sheets and recycled materials, emits considerable amounts of dust into the surrounding environment. This airborne dust poses a significant risk to the respiratory health of workers in the paper industry [7].

Several studies have previously been conducted on respiratory health among workers in the paper industry, though most have focused on the soft paper sector and have been carried out in European countries [8,9,10,11,12]. Moreover, the results from some of these studies are inconclusive [12], or the dust measurement methods used [7,8,9,12] did not adhere to current standards for sampling of health-related fractions of particles [13]. In Ethiopia, recycled paper is primarily used as raw material in the paper industry. There are few studies on respiratory health for this type of industry. However, a recent study from Ethiopia shows that the inhalable paper dust exposure from recycled paper is high, exceeding the value for the Swedish occupational exposure limit in 120 of 150 samples [14]. Therefore, we planned a study of the respiratory health among these workers. With information about the exposure level as well as exposure time of the workers, we would be able to calculate the cumulative dust among them and study a possible dose–response relationship among the exposure and respiratory symptoms. Our aim was to evaluate the potential relationship between cumulative paper dust exposure and respiratory symptoms among workers in the paper industry of Ethiopia.

## 2. Materials and Methods

### 2.1. Study Area, Period, and Design

According to a report by the Ethiopian Chemical Corporation, there are 58 companies in Ethiopia engaged in various aspects of paper production [15]. These activities include paper converting and packaging and the production of soft paper tissues, labeling paper, diapers, and wipes. However, among these, only nine companies focus on the production of paper and paper products. In this study, we selected four paper industry plants, referred to as A (established in 1962), B (2018), C (1997), and D (2015), that utilize imported rolled pulp and recycled paper as primary materials for manufacturing paper and its derivatives. For a comparative analysis, we selected one water bottling industry with five branches in the designated study area as a non-exposed group, as the water bottling production process is designed to be free of dust. This study used a comparative cross-sectional design and was performed from January 2023 to March 2023.

### 2.2. Source and Study Population

The source population comprised all workers employed in the four selected paper industries and one water bottling industry in Ethiopia. The study population included workers engaged in the production processes in both industries. Workers who had been employed in the factory production process for at least one year were included for this study. A minimum of one year was selected as a criterion, because the questionnaires on chronic respiratory symptoms assessed symptoms with durations ranging from one week to three months within the past year.

### 2.3. Sample Size Determination

To determine the sample size for our study, we employed a double population formula, assuming a 1:1 ratio for the exposed and non-exposed groups. Our calculations were based on a previous study conducted among paper industry workers in Ethiopia, where chronic respiratory symptoms varied from 17% to 32.5% in the exposed group and between 4.4% and 8.9% in the non-exposed group [7]. We used a conservative strategy for the estimation by using the smallest difference, i.e., the lowest prevalence in the exposed group (17%) and the highest among the controls (8.9%). We aimed to achieve a statistical power of 95% to detect the difference in proportions between the two groups at a significance level of 0.05. After accounting for a 5% non-response rate, we calculated that 540 participants were required, with 270 from the exposed group and 270 from the non-exposed group.

### 2.4. Data Collection Procedures and Tools

Data were collected by trained data collectors using a standardized questionnaire modified from the American Thoracic Society’s ATS-DLD-78-A to suit the local context [16]. Three data collectors were trained and performed the data collection, with one supervisor regularly present during this fieldwork. The questionnaire template was coded using an open-source software for Computer-Assisted Personal Interviewing using the Census and Survey Processing System (CSPro) (version 7.7.3 [17]) mobile application. The standardized questionnaire was initially developed in English and subsequently translated into the Afan Oromo and Amharic languages for data collection during fieldwork.

The questionnaire included socio-demographic characteristics such as age, sex, and occupational history: working hours per week (in hours); duration of employment in the paper industry (in years); previous work in other dusty industries (yes or no); job groups and years served in each group (preparation, paper machine, finishing, and packing), as well as personal and organizational factors such as health and safety training (yes or no), history of respiratory illnesses (yes or no), family history of respiratory illnesses (yes or no), use of respiratory protective equipment daily (yes or no), cooking inside the home using biofuels (yes or no), and smoking habits (current or former smoker).

Furthermore, the questionnaire included a modified ATS chronic respiratory symptom assessment. Chronic coughs among the participants were identified if they reported “yes” to coughing for most days over a consecutive period of three months within a year. Coughs with sputum/phlegm among the participants were identified if they reported “yes” to coughing with sputum for most days over a consecutive period of three months within a year. Participants were identified as experiencing breathlessness if they reported being troubled by shortness of breath when hurrying on level ground or walking up a slight hill, or if they experienced shortness of breath when walking at their own pace on level ground. Participants were identified as experiencing wheezing if they reported their chest ever sounding wheezy, characterized by a whistling sound.

### 2.5. Exposure Assessment

In a previous publication, we reported personal inhalable dust levels among 150 paper industry workers from the same four paper mills as described in the present study [14]. In short, for each of the four paper mills, ten personal inhalable paper dust samples were taken for four job groups (preparation, paper machine, finishing/converting, and packing) by plastic IOM samplers (SKC Ltd., Blandford Forum, UK), which operated at a flow rate of 2.0 L/min [18]. Our previous study [14] estimated the overall arithmetic mean (AM) of personal inhalable paper dust exposure to be 4.5 mg/m^3^. The AM exposures across the 16 respective job groups ranged from 1.7 mg/m^3^ to 17.8 mg/m^3^. A JEM [19] was developed based on the AM of the paper dust exposure for the four specified job groups (preparation, paper machine, finishing, and packing) within the four paper factories, resulting in 16 exposure combinations.

Most workers had worked in more than one department over the study period. For each worker, the cumulative dust exposure, in mg·year/m^3^, was calculated as the sum of the products of the department’s AM exposure to inhalable paper dust (mg/m^3^) and the number of years employed in that specific department.

The water bottling industry was assumed to have a low dust exposure level. Thus, workers from this industry were used as a non-exposed group for chronic respiratory symptoms. In line with this, a previous study in Ethiopia reported that the dust level in the bottling industry was measured to be very low, with an AM of 0.33 mg/m^3^ (range of 0.11–1.16 mg/m^3^) [20]. One of the water bottling factories from this previous study is in our current study area, and it is likely that this water bottling factory, in our present study, still has a similarly low dust exposure level.

### 2.6. Data Management and Analysis

The principal investigator closely monitored the data collection process to ensure completeness and consistency of the data. Data were collected using CSPro version 7.7.3 (Census Bureau, Washington, DC, USA) and later exported to SPSS version 26 (IBM Corp., Armonk, NY, USA) for analysis.

We used descriptive statistics, including Pearson’s chi-square test or Fisher’s exact test when the expected value was less than 5, to test differences in categorical variables. Independent *t*-tests were used to compare the mean value of continuous variables. A Poisson regression analysis with robust variance was used to determine the prevalence ratio (PR) of chronic coughs, phlegm, wheezing, and breathlessness, respectively, between groups of workers, while adjusting for sex, age, history of respiratory symptoms, history of dust exposure in other industries, and cooking food at home using biofuels. As there were only a few ever-smokers, smoking was not included in the regression analysis. The variables included in the regression analysis were chosen based on a significance level of *p* < 0.05 in a univariate analysis or based on whether they could be considered logical regarding the expected impact on respiratory symptoms. The prevalence ratio was more suitable than the prevalence odds ratio, because the odds ratio overestimates the strength of associations when the prevalence is greater than 10% [21,22].

First, a Poisson regression analysis was utilized to compare the PR of chronic respiratory symptoms between paper industry workers and non-exposed groups with a 95% confidence interval and a statistical significance of *p* < 0.05. Secondly, a Poisson regression analysis was used to determine the exposure–response relationship between cumulative dust exposure and chronic respiratory symptoms among paper industry workers, while adjusting for the same factors as indicated above. Although age and cumulative dust correlated significantly (r = 0.78 and *p* < 0.001), we still included age in this regression analysis, since both these variables may impact respiratory symptoms.

## 3. Results

### 3.1. Socio-Demographic and Personal Characteristics of Participants

A total of 537 individuals participated in the study, with 270 in the exposed group and 267 in the non-exposed group, yielding a response rate of 99.4%. Most participants were male, with 60.0% (162) in the exposed group and 70.4% (188) in the non-exposed group. The exposed group was significantly older than the non-exposed group (mean: 32 vs. 26 years) and had worked for more years (mean: 9 vs. 2 years). The working hours per week were higher among the non-exposed than the exposed group (52 vs. 48 h) (Table 1). There were no differences between the groups regarding the number of years they had worked in other dusty industries, previous respiratory diseases, cook food at home using biofuels, receiving health and safety training, and using personal protective equipment (PPE). Only a few workers in the exposed group were current (1.1%) or ever-smokers (1.5%), with no difference between the groups, and for these two variables, the *p* values were not considered due to low numbers (Table 1).

Overall, the AM cumulative dust exposure among the 270 paper industry workers was estimated to be 31.6 mg·year/m^3^, ranging from 2.5 mg·year/m^3^ to 134.1 mg·year/m^3^. The cumulative dust exposure was higher in the two oldest paper mills, Factory A (AM = 53.5 mg·year/m^3^ with a range of 2.5–34.1 mg·year/m^3^) and Factory C (24.5 and 8.5–68.0), than in the two newest paper mills: Factory B (12.9 and 3.5–57.0) and Factory D (11.0 and 2.5–28.8). Cumulative dust exposure correlated strongly with both age (r = 0.78 and *p* < 0.001) and employment duration (r = 0.94 and *p* < 0.001) and correlated moderately with hours worked per week (r = −0.38 and *p* < 0.001).

### 3.2. Prevalence Ratio of Chronic Respiratory Symptoms

The prevalence of all four chronic respiratory symptoms studied was significantly higher among paper industry workers than among water bottling industry workers (Table 2): chronic coughs (27.4% vs. 7.5%), coughs with sputum/phlegm (21.1% vs. 1.1%), breathlessness (25.6% vs. 11.0%), and wheezing (25.6% vs. 5.0%) (Table 2).

Our findings reveal a higher prevalence ratio of chronic coughs (APR = 3.3 and 95% CI: 2.0–5.4), breathlessness (APR= 2.2 and 95% CI: 1.4–3.4), and wheezing (APR = 4.3 and 95% CI: 2.3–7.7) among paper industry workers than those in the water bottling industry, after adjusting for sex, age, working in other dusty industries, previous respiratory illnesses, and cooking food at home using biofuels (Table 2). The variable coughs with sputum/phlegm was not analyzed due to the low number of respondents with this symptom in the non-exposed group.

The multiple regression analysis indicated that the risk of wheezing increased with increasing age, while workers who had been exposed to dusty industries before were 2.3 times more likely to develop chronic coughs and 1.8 times more likely to experience wheezing compared to those who had not been exposed to other dusty workplaces. Additionally, having a history of respiratory illnesses was significantly associated with increased chronic coughs (Table 2).

When including only the paper factory workers, the multiple regression analysis indicated a significant relationship between cumulative dust exposure and chronic coughs when adjusting for age, sex, dust exposure history in other industries, previous history of respiratory illnesses, and cooking food using biofuels.

## 4. Discussion

This study revealed a significantly higher prevalence ratio of chronic respiratory symptoms among workers exposed to paper dust than unexposed workers in the water bottling industry. The prevalence of chronic respiratory symptoms among the exposed group ranged from 21% to 27%, in contrast to the non-exposed group, which ranged from 1% to 11%. A significant relationship between cumulative dust exposure and chronic coughs was found among workers in the paper industry.

The higher prevalence ratio of chronic respiratory symptoms among paper industry workers than the non-exposed workers suggests that individuals exposed to paper dust are at a higher risk of developing respiratory symptoms compared to those who are not exposed to paper dust. These findings align with previous studies conducted in the paper and soft tissue industries, both in Ethiopia and Sweden, indicating that exposure to paper dust may be a significant risk factor for chronic respiratory symptoms [7,23,24].

However, the current study found a lower occurrence of chronic respiratory symptoms compared to a similar study carried out in Ethiopia [7]. This difference in findings could be attributed to the inclusion of relatively newly established factories in the present study, Factory B and D [14], where the workers had a lower cumulative dust exposure, compared to those in the two older factories that were part of a previous study [7]. Although care should be taken when comparing studies, the prevalence of chronic coughs in our study (27.4%) seems to be higher than among workers exposed to the highest cumulative dust levels in the soft tissue paper industry in Germany (17.2%) [8]. These variations could be partly due to differences in the types of raw materials used. The soft tissue paper mill utilizes cellulose as raw materials, whereas the mill investigated in the present study primarily uses recycled paper. Additionally, the German study focused solely on the paper machine and combined working job groups, while the present study encompassed all production workers, including those involved in the paper machine, preparation, finishing, and packing job groups.

It is important to note that other cross-sectional studies conducted in Croatia and Sweden [11,25] reported a higher prevalence of chronic respiratory symptoms than the current study. These variations could be attributed to factors such as the exclusive use of recycled paper as raw materials, the relatively small sample size, and a majority of the participants being cigarette smokers in the Croatian study [11]. A review study involving different countries [25] focused on the oldest mills, where wood fibers are used as raw materials, and exposure to chlorine, chlorine dioxide, terpenes, sulfur dioxide, and paper dust was considered for the development of chronic respiratory symptoms. However, for the mills investigated in our study, recycled paper is primarily used as raw materials.

In the present study, the risk of chronic coughs increased by 1.2% with each unit increase in cumulative dust exposure. This finding indicates an exposure–response relationship between the amount of dust inhaled over time (mg·year/m^3^) and the development of chronic coughs. This relationship remained after controlling for sex, age, prior respiratory illnesses, exposure to dust in other industries, and cooking with biofuels at home. Since age is considered a major risk factor for developing chronic respiratory symptoms [26], we chose not to exclude it from the multivariate analysis, although a univariate analysis showed a significant correlation between cumulative dust exposure and age. This decision was based on the understanding that as individuals age, there is a noticeable decline in the functioning of their respiratory system, partly because of exposure but also due to multiple factors other than paper dust [27]. The cumulative dust exposure was explained in terms of the years of employment in the paper industry multiplied by the mean exposure level of the respective job groups, indicating that the employment duration and cumulative exposure to dust tend to increase with age.

A cough was the only symptom showing a significant relationship with cumulative dust. A cough may also be an acute, irritative symptom, being prominent due to a persistent immune response. A previous study reported a significant relationship between cumulative dust and impaired lung function in workers exposed to the total paper dust in soft tissues [9], but this study did not find any relationship between dust exposure and chronic bronchitis among the workers, and the cough symptoms were not analyzed specifically. Additionally, a study conducted in Germany in the soft tissue industry identified no significant association between cumulative dust exposure and respiratory symptoms [8].

The current study also indicated that other factors, including sex, were significantly associated with chronic respiratory symptoms. This finding is consistent with previous studies [7,8,9]. Furthermore, older workers were more prone to develop wheezing than workers of lower age, which was also shown in a previous study [7].

### The Strengths and Limitations of This Study

A strength of this study is that we used a non-exposed group for the comparative study and a sampler for the measurements of personal inhalable paper dust. Additionally, we developed a JEM to estimate the exposure–response relationship by calculating the cumulative dust for each worker. We accounted for variations in exposure across job groups, as most workers were classified into more than one exposure category over their work history in the paper industry. However, a limitation of this study was our assumption that the exposure level of paper dust remained constant over time within the respective job groups when calculating the cumulative dust exposure. Another limitation of this study was its cross-sectional design.

This study also relied on self-reports, which is a weakness. Although we used a standardized questionnaire designed to capture chronic symptoms, we cannot be sure that the workers did not report acute, irritative symptoms instead. Future studies should include objective examinations of respiratory health. Additionally, other confounding factors, such as infections, which were not examined in this study, may also have contributed to the recorded symptoms among the workers. Despite these considerations, the high dust levels measured in this study likely played a significant role in the observed respiratory symptoms.

A significant weakness of this study of the effects of cumulative dust on respiratory health among the exposed workers is the correlation between age and exposure to dust. Older workers tend to have a longer duration of exposure, leading to higher cumulative dust levels. This correlation makes it challenging to separate the effects of dust exposure from the natural decline in respiratory function that typically accompanies aging. As a result, it becomes difficult to determine whether the observed respiratory issues among paper industry workers are due to prolonged dust exposure, age-related respiratory decline, or a combination of both factors.

We recommend that future researchers conduct longitudinal studies to establish a more definitive exposure–response relationship between cumulative dust exposure and respiratory health. Also, the number of workers in some subgroups of this study was low, suggesting the need for a larger study population.

## 5. Conclusions

Workers in the paper industry have a higher occurrence of respiratory symptoms than those employed in the water bottling industry. The findings indicate a significant association between dust exposure and coughing, but this finding has uncertainties. However, together with the high dust levels found, based on these findings, we suggest that employers implement engineering control measures to mitigate the development of respiratory symptoms among workers in the paper industry.

## Figures and Tables

**Table 1:** Socio-demographic and personal characteristics of exposed (paper industry) and non-exposed (water bottling industry) workers in Ethiopia, 2023.

Variables	Categories	Exposed (n = 270)	Non-Exposed (n = 267)	*p* Value
Age (years); Mean (range)		32 (18–68)	26 (18–53)	<0.001 ^a^
Hours per week; Mean (range)		48 (40–56)	52 (40–72)	<0.001 ^a^
Employment duration (years); Mean (range)		9 (1–45)	2 (1–8)	<0.001 ^a^
Sex; n (%)	Male Female	162 (60.0) 108 (40.0)	188 (70.4) 79 (29.6)	0.014 ^b^
Worked in other dusty industries; n (%)	No Yes	244 (90.4) 26 (9.6)	242 (90.6) 25 (9.4)	1.00 ^b^
Previous respiratory illnesses; n (%)	No Yes	258 (95.6) 12 (4.4)	260 (97.4) 7 (2.6)	0.351 ^b^
Ever smoked cigarettes; n (%)	No Yes	266 (98.5) 4 (1.5)	267 (100) 0	nd
Currently smoking cigarettes; n (%)	No Yes	267 (98.9) 3 (1.1)	267 (100) 0	nd
Cook food at home using biofuels; n (%)	No Yes	53 (19.6) 217 (80.4)	41 (15.4) 226 (84.6)	0.212 ^b^
Health and safety training; n (%)	No Yes	163 (60.4) 107 (39.6)	169 (63.3) 98 (36.7)	0.534 ^b^
Wear always RPD at work; n (%)	No Yes	204 (75.6) 66 (24.4)	212 (79.4) 55 (20.6)	0.303 ^b^

^a^ Independent *t*-test, ^b^ Pearson’s chi-square test, n = numbers, nd = not determined, RPD = respiratory protective device.

**Table 2:** Prevalence (in %) and adjusted prevalence ratio (APR) of chronic respiratory symptoms among (**A**) exposed paper industry and non-exposed bottling industry workers (n = 537) and (**B**) exposed paper industry workers (n = 270) in Ethiopia, 2023. Adjusted for sex, age, worked in other dusty industries, previous respiratory illnesses, and cooking food at home using biofuels.

Variables	Categories	(A) Chronic Respiratory Symptoms among Paper and Water Bottling Industry Workers (n = 537)							
		Chronic Coughs		Coughs with Sputum/Phlegm		Breathlessness		Wheezing	
		n (%)	APR (95% CI)	n (%)	APR (95% CI)	n (%)	APR (95% CI)	n (%)	APR (95% CI)
Exposure status	Non-exposed Exposed	20 (7.5) 74 (27.4) ^a,^*	1.0 3.3 (2.0–5.4)	3 (1.1) 57 (21.1) ^a,^*		29 (11.0) 69 (25.6) ^a,^*	1.0 2.2 (1.4–3.4)	13 (5.0) 69 (25.6) ^a,^*	1.0 4.3 (2.3–7.7)
Sex	Male Female	67 (19.1) 27 (14.4) ^a^	1.00 0.8 (0.5–1.2)	43 (12.3) 17 (9.1) ^a^		58 (16.6) 40 (21.1) ^a^	1.00 1.2 (0.8–1.8)	51 (14.6) 31 (16.6) ^a^	1.00 1.2 (0.8–1.9)
Age in years	Continuous variable		1.02 (0.99–1.04)				1.01 (0.90–1.03)		1.03 (1.01–1.05)
Worked in other dusty industries	No Yes	75 (15.4) 19 (37.3) ^a,^*	1.00 2.3 (1.5–3.4)	54 (11.1) 6 (11.8) ^a^		91 (18.7) 7 (13.7) ^a^	1.00 0.8 (0.4–1.5)	70 (14.4) 12 (23.5) ^a^	1.00 1.8 (1.1–3.0)
Previous respiratory illnesses	No Yes	85 (16.4) 9 (47.3) ^c,^*	1.00 2.4 (1.5–3.8)	51 (9.8) 9 (47.4) ^c,^*		93 (18.0) 5 (26.3) ^c^	1.00 1.3 (0.6–2.8)	77 (14.9) 5 (26.3) ^c^	1.00 1.4 (0.7–2.7)
Cook food at home using biofuels	No Yes	20 (21.3) 74 (16.7) ^a^	1.00 0.9 (0.6–1.5)	18 (19.1) 42 (9.5) ^a,^*		20 (21.3) 78 (17.6) ^a^	1.00 0.8 (0.5–1.3)	17 (18.1) 65 (14.7) ^a^	1.00 0.9 (0.6–1.5)
**(B) Chronic Respiratory Symptoms among Workers Exposed to only Personal Inhalable Paper Dust (n = 270)**									
Cumulative dust (mg/m^3^ × years)	AM (SD)	41.1 (35.7) ^b,^*	1.012 (1.003–1.022)	44.1 (38.5) ^b,^*	1.01 (0.99–1.02)	34.0 (30.9) ^b^	1.00 (0.99–1.01)	38.0 (31.3) ^b,^*	1.01 (0.99–1.01)
Sex	Male Female	55 (34.0) 19 (17.6) ^a,^*	1.00 0.6 (0.3–0.9)	42 (25.9) 15 (13.9) ^a,^*	1.00 0.6 (0.3–1.1)	42 (25.9) 27 (25.0) ^a^	1.00 0.9 (0.6–1.5)	41 (25.3) 28 (25.9) ^a^	1.00 1.2 (0.8–2.0)
Age in years	Continuous variable		0.98 (0.95–1.01)		1.00 (0.97–1.03)		0.99 (0.96–1.03)		1.02 (0.99–1.05)
Worked in other dusty industry	No Yes	61 (25.0) 13 (50.0) ^a,^*	1.00 2.1 (1.3–3.2)	51 (20.9) 6 (23.1) ^a^	1.00 1.1 (0.6–2.3)	62 (25.4) 7 (26.9) ^a^	1.00 1.2 (0.6–2.1)	59 (24.2) 10 (38.5) ^a^	1.00 1.8 (1.1–3.3)
Previous respiratory illnesses	No Yes	66 (25.6) 8 (66.7) ^c,^*	1.00 2.3 (1.4–3.7)	48 (18.6) 9 (75.0) ^c,^*	1.00 3.5 (2.2–5.6)	66 (25.6) 3 (25.0) ^c^	1.00 0.9 (0.4–2.5)	64 (24.8) 5 (41.7) ^c^	1.00 1.6 (0.8–3.2)
Cook food at home using biofuels	No Yes	19 (35.8) 55 (25.3) ^a^	1.00 0.9 (0.6–1.5)	18 (34.0) 39 (18.0) ^a,^*	1.00 0.8 (0.5–1.3)	12 (22.8) 57 (26.3) ^a^	1.00 1.2 (0.7–2.1)	14 (26.4) 55 (25.3) ^a^	1.00 0.9 (0.67–1.7)

^a^ Pearson’s chi-square test, ^b^ Independent *t*-test, ^c^ Fisher’s exact test, * *p* value < 0.05.

## Data Availability

The raw data supporting the conclusions of this article will be made available by the authors on request.
